# Assessment of quality of life in individuals with chronic headache. Psychometric properties of the WHOQOL-BREF

**DOI:** 10.1186/s12883-020-01845-7

**Published:** 2020-07-03

**Authors:** Patrick Brzoska

**Affiliations:** Health Services Research, Faculty of Health, School of Medicine, Witten/Herdecke University, Alfred-Herrhausen-Straße 50, D-58448 Witten, Germany

**Keywords:** Quality of life, Assessment, Differential item functioning, WHOQOL-BREF

## Abstract

**Background:**

The WHOQOL-BREF is a frequently used instrument for the assessment of health-related quality of life. Unlike other generic instruments used for the assessment of this construct, little is known about its properties in individuals with headache disorders. The present study examines the reliability and factorial validity of the WHOQOL-BREF in individuals with chronic headache residing in Austria.

**Methods:**

Data from a representative population-based survey on 963 individuals with chronic headache surveyed between 2013 and 2015 was used. The factorial validity was examined by means of confirmatory factor analysis. Differential item functioning related to sex was analyzed using multiple indicators multiple causes models.

**Results:**

Information on 239 men and 724 women with chronic headache was available. The four-factor, 24-item baseline model showed a moderate fit (RMSEA = 0.066; CFI = 0.868; TLI = 0.852; SRMR = 0.053), which improved significantly after the addition of six error covariances (RMSEA = 0.052; CFI = 0.920; TLI = 0.908; SRMR = 0.046). Sex-related differential item functioning was observed in two items of the environment factor, two items of the psychological health factor and two items of the physical health factor.

**Conclusions:**

After some modifications to the measurement model, the WHOQOL-BREF shows a satisfactory fit among individuals with chronic headache in Austria. Because of these modifications and the questionnaire’s susceptibility for differential item functioning, a latent variable framework should be employed for the analysis. Future studies need to confirm these results for other language regions and should also examine different subtypes of headache.

## Background

Health-related quality of life (HRQOL) is an important patient-reported outcome in health research and practice. It is used as an indicator in clinical trials, in the evaluation of health care services and for benchmarking purposes. Differences in HRQOL between population groups can help to identify deficits in health care and to guide measures aiming to reduce disparities [1]. Also for headache disorders the measurement of HRQOL is necessary to assess how headaches affect individuals in their daily activities and to evaluate the effectiveness of therapeutic regimens, thus being indispensable to ensure patient-centered health care. In previous studies, for example, HRQOL has been used as an outcome to study the effectiveness of pharmaceutical [2] and psychological interventions [3] in patients with migraine or to examine how headache disorders are related to well-being in different population groups [4].

An important requirement for the use of HRQOL as an indicator in research and practice is the valid and reliable assessment of this construct. Comparable to other psychological constructs, HRQOL is usually assessed by means of multi-item self- or interviewer-administered questionnaires where different latent dimensions (‘factors’) are measured by a set of observable items (‘indicators’) [5]. Despite focusing on quality of life in general, the short version of the World Health Organization Quality of Life (WHOQOL) questionnaire (WHOQOL-BREF) is one of the most frequently used generic instruments applied for this purpose [6]. Based on the 100-item WHOQOL [7], the WHOQOL-BREF consists of 24 Likert scale items on different facets of HRQOL (such as pain, sleep, self-esteem and sexual activity) which represent four latent HRQOL dimensions: physical health, psychological health, social relationships and environment. Two additional items, which are not part of the aforementioned measurement model, assess individuals’ satisfaction with life and their overall quality of life. The WHOQOL-BREF uses a 5-point Likert response format with six sets of response categories (e.g., 1 “Not at all” to 5 “completely” or 1 “Very dissatisfied” to 5 “Very satisfied”; see Table 2 for response categories by items). With the exception of three inversely coded items, a higher score on each item indicates a higher quality of life with respect to the facet the item is measuring. In substantive research, the WHOQOL-BREF is either examined based on the four aforementioned dimensions (e.g., [8–11]) or these dimensions are summarized into a global quality of life score, effectively extending the four-factor measurement model into a second-order model (e.g., [12–15]).

The WHOQOL-BREF has been frequently used to examine the HRQOL of patients with different chronic conditions, including headache disorders [16–19]. Although previous studies have shown that the WHOQOL-BREF can be applied across different diagnostic groups [20], some studies have indicated that modifications to the measurement model of the WHOQOL-BREF may be necessary to achieve sufficient model fit [21–23]. Research has also indicated that some items may be prone to differential item functioning related to sociodemographic variables such as age and sex [24–26]. Differential item functioning refers to the situation in which items perform differently across population groups despite the underlying dimensions these items are purported to measure are held constant. This for example means that men and women with the same level of HRQOL may a have different probability for a certain item response [27].

Whereas the performance and psychometric properties of other generic HRQOL assessment instruments such as the SF-36 and the EQ-5D have been examined among individuals with headache disorders [28], little is known about the validity of the WHOQOL-BREF in this respect. Confirming the validity of the WHOQOL-BREF in individuals with headache disorders could further promote the use of quality of life as a quality indicator in headache care [29, 30]. Extending own previous research on the subject [31], the aim of the present study was to examine the reliability and factorial validity of the WHOQOL-BREF in a representative population-based sample of individuals with self-reported chronic headache residing in Austria and to assess its measurement equivalence between men and women.

## Methods

### Data and variables

Data from a representative cross-sectional population-based health survey conducted in Austria between 2013 and 2015 (‘Austrian Health Interview Survey 2014’) was used providing information on 963 respondents with self-reported chronic headache. The German-language anonymous and voluntary survey was carried out by the Austrian statistical office (‘Statistics Austria’) by means of computer-assisted telephone interviewing. Its implementation is part of the health reporting activities which Statistics Austria is routinely conducting and fulfils all requirements and guidelines of the Federal Statistics Act. Survey participants provided informed consent prior to their participation. Researchers can obtain the data used in the present study free of charge from Statistics Austria [32].

Aside from the 24 items of the WHOQOL-BREF, information on *age* (15–29 years, 30–44 years, 45–59 years, 60+ years), *sex*, *partnership status* (living in a partnership, not living in a partnership), *educational level* (primary/lower secondary, upper secondary/post-secondary [non-tertiary], tertiary education [bachlor, master, doctoral]) and *net equivalence income* (quantiles) were used for purposes of sample description in the present study. Respondents’ education was measured by means of eight categories following the International Standard Classification of Education (ISCED) [33].

### Statistical analysis

χ^2^-tests were calculated for purposes of sample description (Table 1). For each of the 24 items of the WHOQOL-BREF measurement model also means, standard deviations (sd), skewness and kurtosis have been calculated, and the distribution of the items has been examined graphically be means of histograms. In addition to skewness and kurtosis, also the results of omnibus normality tests based on these two measures are reported [34]. Multivariate normality has been examined by means of the Henze-Zirkler test [35]. A correlation matrix of the 24 items is provided in Additional file 1. The dataset had no values missing.

**Table 1:** Description of the study sample by sex (individuals with self-reported chronic headache residing in Austria, Austrian Health Interview Survey, 2013–2015, *n* = 963)

	Sex		*p*-value*
	Male	Female	
**N**	239	724	
**Age**			0.22
15–29 years	28 (11.7%)	84 (11.6%)	
30–44 years	72 (30.1%)	263 (36.3%)	
45–59 years	91 (38.1%)	265 (36.6%)	
60+ years	48 (20.1%)	112 (15.5%)	
**Partnership status**			0.80
Living in a partnership	150 (62.8%)	461 (63.7%)	
Not living in a partnership	89 (37.2%)	263 (36.3%)	
**Net equivalence income of respondent’s household**			0.34
Below 1st quantile	64 (26.8%)	183 (25.3%)	
Between 1st and 2nd quantile	48 (20.1%)	177 (24.4%)	
Between 2nd and 3rd quantile	41 (17.2%)	146 (20.2%)	
Between 3rd and 4th quantile	49 (20.5%)	129 (17.8%)	
Between 4th and 5th quantile	37 (15.5%)	89 (12.3%)	
**Educational level**			0.01
Primary/lower secondary	42 (17.6%)	189 (26.1%)	
Upper secondary/post-secondary (non-tertiary)	152 (63.6%)	389 (53.7%)	
Tertiary education (bachlor, master, doctoral)	45 (18.8%)	146 (20.2%)	

*Note.* Because of rounding not all percentages add up to 100%. * *p*-value from chi-square test

Given that the items were not normally distributed as becomes evident from the skewness and kurtosis values (Table 2) as well as the respective tests for univariate and multivariate normality (*p* < 0.001), robust maximum likelihood (MLR) confirmatory factor analysis (CFA) was used to examine the factorial validity of the WHOQOL-BREF [36]. The standard measurement baseline model tested by means of CFA comprised the physical health (7 items), the psychological health (6 items), the social relationships (3 items) and the environment (8 items) factor [6]. In addition, the second-order factor measurement model, in which the four domains are conceptualized to be influenced by a higher-order dimension (‘global quality of life’) [6], was tested, considering that this measurement model is also frequently applied in substantive research. Differential item functioning related to gender was analyzed by means of multiple indicators multiple causes (MIMIC) models following established guidelines [36].

**Table 2:** Descriptive statistics of the 24 items of the WHOQOL-BREF measurement model (individuals with self-reported chronic headache residing in Austria, Austrian Health Interview Survey, 2013–2015, *n* = 963)

Item number^**a**^	Item content	Mean	sd	Skewness	Kurtosis	***p***-value^**b**^
3.	To what extent do you feel that physical pain prevents you from doing what you need to do?^1^	2.36	1.20	0.52	2.24	< 0.001
4.	How much do you need any medical treatment to function in your daily life?^1^	2.07	1.26	0.90	2.55	< 0.001
5.	How much do you enjoy life?^1^	3.60	0.88	−0.40	2.99	< 0.001
6.	To what extent do you feel your life to be meaningful?^1^	4.03	0.96	−0.96	3.59	< 0.001
7.	How well are you able to concentrate?^2^	3.72	0.84	−0.51	3.29	< 0.001
8.	How safe do you feel in your daily life? ^2^	3.90	0.80	−0.68	3.90	< 0.001
9.	How healthy is your physical environment? ^2^	3.93	0.83	−0.69	3.92	< 0.001
10.	Do you have enough energy for everyday life?^3^	3.61	0.89	−0.32	2.80	< 0.001
11.	Are you able to accept your bodily appearance?^3^	3.89	0.92	−0.69	3.41	< 0.001
12.	Have you enough money to meet your needs?^3^	3.31	1.11	−0.24	2.40	< 0.001
13.	How available to you is the information that you need in your day-to-day life?^3^	4.19	0.86	−0.93	3.44	< 0.001
14.	To what extent do you have the opportunity for leisure activities?^3^	3.66	1.07	−0.38	2.35	< 0.001
15.	How well are you able to get around?^4^	4.19	0.90	−0.98	3.46	< 0.001
16.	How satisfied are you with your sleep?^5^	3.44	1.18	−0.50	2.32	< 0.001
17.	How satisfied are you with your ability to perform your daily living activities?^5^	3.84	0.98	−0.85	3.31	< 0.001
18.	How satisfied are you with your capacity for work?^5^	3.74	1.13	−0.91	3.07	< 0.001
19.	How satisfied are you with yourself?^5^	3.86	0.95	−0.86	3.45	< 0.001
20.	How satisfied are you with your personal relationships?^5^	4.09	0.96	−1.14	4.12	< 0.001
21.	How satisfied are you with your sex life?^5^	3.56	1.09	−0.71	2.98	< 0.001
22.	How satisfied are you with the support you get from your friends?^5^	3.90	0.93	−1.03	4.27	< 0.001
23.	How satisfied are you with the conditions of your living place?^5^	4.18	0.93	−1.23	4.31	< 0.001
24.	How satisfied are you with your access to health services?^5^	3.98	0.89	−1.06	4.38	< 0.001
25.	How satisfied are you with your transport?^5^	3.87	0.99	−0.93	3.66	< 0.001
26.	How often do you have negative feelings such as blue mood, despair, anxiety, depression?^6^	2.63	1.00	0.32	2.55	< 0.001

*Note.*^a^The numbering is in accordance with the item sequence of the WHOQOL-BREF, of which only items 3 to 26 are part of the measurement model (see Methods section). ^b^p-value from D’Agostino and Pearson’s chi-square omnibus test for normality. Response format: ^1^″Not at all” to “An extreme amount”; ^2^″Not at all” to “Extremely”; ^3^″Not at all” to “Completely”; ^4^“Very poor” to “Very good”; ^5^“Very dissatisfied” to “Very satisfied”; ^6^″Never” to “always”

The fit of the measurement model was examined by means of the Tucker-Lewis index (TLI), the comparative fit index (CFI) and the standardized root mean square residual (SRMR) with TLI and CFI values > 0.90 and SRMR values ≤0.08 considered to indicate acceptable model fit. In addition, the root mean square error of approximation (RMSEA) was calculated with values ≤0.06 considered indicating a good model fit [37, 38]. To identify potential for model improvement, modification indices were calculated. Only theoretically sound modifications were implemented [36].

The reliability was assessed by means of composite reliability estimates based on the factor loadings estimated by the CFA model. Estimates ≥0.70 were considered to indicate acceptable reliability in the latent dimensions [39]. In addition, Cronbach’s alpha estimates were calculated. Given its limitations [36, 40], however, these estimates should only be interpreted with caution and are presented in this study mainly to facilitate comparisons with previous research that relied on that measure.

The analyses were performed by means of Stata 15 [41] and the R package lavaan 0.6–3 [42].

## Results

Information on 239 men and 724 women with a chronic headache was available. Both groups did not differ from each other in terms of age, the proportion of individuals living with a partner and net equivalence income. Women had a slightly lower educational level, with a higher proportion of women having only a primary or lower secondary education (Table 1).

**Fig. 1:**
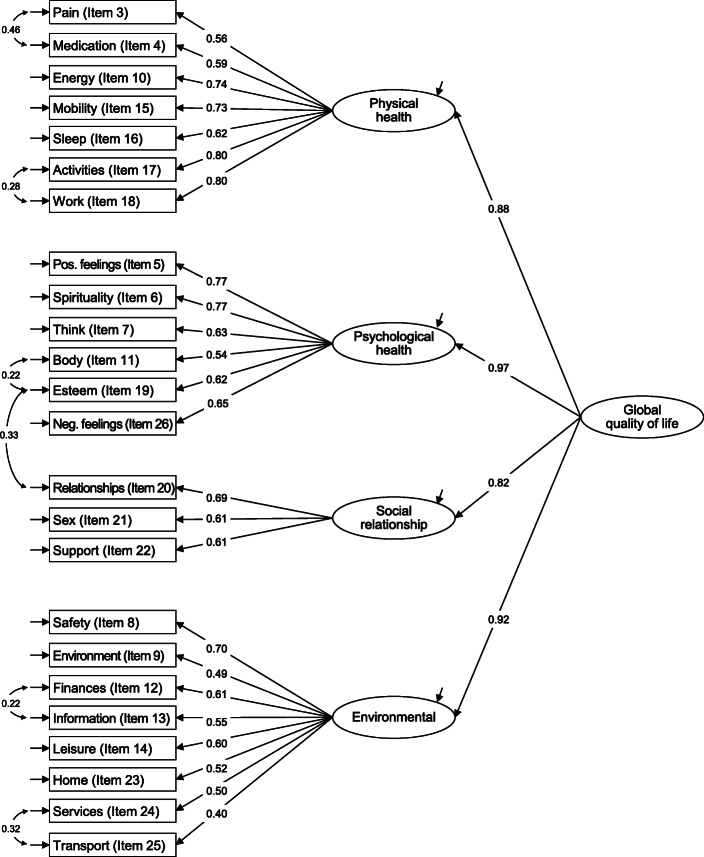
Factor structure of the WHOQOL-BREF in individuals with self-reported chronic headache residing in Austria (numbers displayed on the straight and curved arrows signify completely standardized factor loadings and covariances, respectively; Austrian Health Interview Survey, 2013–2015, *n* = 963; All factor loadings/covariances were significant at *p* < 0.001)

The four-factor, 24-item baseline model showed a moderate fit (χ^2^ = 1290.309, degrees of freedom [df] = 246, *p* < 0.001; RMSEA = 0.066; CFI = 0.868; TLI = 0.852; SRMR = 0.053). After the addition of six error covariances between items 3 and 4 and 17 and 18 of the physical health factor, items 11 and 19 of the psychological health factor, items 19 and 20 of the psychological health and social relationships factor, respectively, and items 12 and 13 and 24 and 25 of the environment factor (Fig. 1) the model fit improved significantly (χ^2^ = 873.394, df = 240; *p* < 0.001; RMSEA = 0.052; CFI = 0.920; TLI = 0.908; SRMR = 0.046). Two items of the environment factor (9 and 25) had low completely standardized factor loadings (λ) of 0.49 and 0.40 respectively; all other factor loadings were of acceptable size (λ ≥ 0.5). All factor loadings were significant at *p* < 0.001. The fit of the second-order measurement model did not differ from the first-order model (χ^2^ = 879.429, df = 242, p < 0.001; RMSEA = 0.052; CFI = 0.919; TLI = 0.908; SRMR = 0.046).

Composite reliability estimates for the WHOQOL-BREF physical, psychological, social relationships and environment factor were 0.87, 0.84, 0.67 and 0.77, respectively. The respective Cronbach’s alpha values were 0.83, 0.80, 0.64 and 0.77, respectively.

Differential item functioning related to sex was observed in items 9 and 14 of the environment factor, items 11 and 26 of the psychological health factor and items 15 and 18 of the physical health factor as evidenced by significant direct effects of sex on these items while holding the respective factors constant. The effects (β = − 0.068, β = − 0.054, β = − 0.108, β = 0.096, β = 0.076 and β = 0.084, respectively), however, were small in size and did not bias the comparison between men and women. Irrespective of adjusting for DIF related to sex, no significant difference in quality of life was identified between men and women.

## Discussion

The WHOQOL-BREF is a frequently used instrument for the assessment of HRQOL. Although applied for the assessment of HRQOL among individuals with headache disorders [16–19], little is known about its psychometric properties. The present study examined the reliability and factor structure of the questionnaire in individuals with chronic headache in Austria and its equivalence between men and women.

The analysis showed that the physical health, psychological health and environment factor of the WHOQOL-BREF had a satisfactory internal consistency. The internal consistency of the social relationships factor was below the recommended threshold of Cronbach’s alpha ≥0.7. Although meta-analytical results are inconsistant [43], evidence suggests that Cronbach’s alpha tends to be smaller for factors with fewer items. Given that the social relationships factor consists only of three items, this could explain its low internal consistency as compared to the other factors identified in this study as well as previous research [44, 45]. The respective composite reliability estimate was slightly larger, however still below the threshold of 0.7. This may indicate some general limitation of the social relationships dimension, which also previous studies had pointed to [13, 46–49].

The present analysis further revealed that the standard WHOQOL-BREF measurement model only showed a moderate fit among individuals with chronic headache in Austria. The fit improved significantly in a reparameterized model after the addition of six error covariances. Five of these covariances concerned items of the same factor, while one error covariance was between items belonging to different factors each (item 19: “How satisfied are you with yourself?” and item 20: “How satisfied are you with your personal relationships?”). It can be assumed that the latter error covariance results from the items being presented subsequently and their conceptual similarity. Also previous research conducted on the factor structure of the WHOQOL-BREF in other population groups suggested that adding error covariances is necessary to improve model fit [22, 50]. Although the addition of these error covariances followed theoretical considerations and the error covariances added were similar to those in previous research, these post hoc modifications applied to the model have to be considered an exploratory type of examination and should, therefore, be cross-validated in other populations.

With the aforementioned modifications implemented, the WHOQOL-BREF can be considered a valid instrument for the assessment of HRQOL in individuals with chronic headache. However, as the analysis has shown, some items are prone to DIF related to sex. This corresponds to findings from research that has been conducted in other settings [44, 51]. Although in the present study DIF was small in size, this potential bias, in general, needs to be taken into account to ensure valid estimates when comparing HRQOL between males and females. Latent variable modeling provides a valuable approach for this purpose and also allows to take into account the aforementioned modifications in terms added error covariances [36].

To the best of the author’s knowledge, this is the first study which examines the psychometric properties of the WHOQOL-BREF in a population with headache disorders. Strength of the present study are its large and nationwide sample as well as the high quality of the data collection [32]. Limitations particularly concern its narrow focus on the population in Austria and chronic headache in general. Future studies should further examine whether the results of the present investigation are also applicable to the study of the HRQOL of individuals with different subtypes of headache and of those who live in other language regions [52]. Furthermore, also other domains of validity of the WHOQOL-BREF in individuals with headache disorders, such as content, convergent and divergent validity need to be explored. Finally, the sources of model ill-fit identified in this study as well as in previous investigations could indicate some general problems of the WHOQOL-BREF measurement model, which should be further investigated in future research, both on headache disorders as well as on other conditions.

## Conclusion

The WHOQOL-BREF is frequently used for the assessment of HRQOL among individuals with chronic conditions. The present study shows that after some modifications the WHOQOL-BREF can also be considered valid for the assessment of HRQOL among individuals with chronic headache. Because of these modifications and the questionnaire’s susceptibility for differential item functioning, a latent variable framework should be employed for the analysis.

## Supplementary information

**Additional file 1 **Correlations between the 24 WHOQOL-BREF items (individuals with self-reported chronic headache residing in Austria, Austrian Health Interview Survey, 2013–2015, *n* = 963)

## Data Availability

Data used in the present study is available for researchers as a Scientific Usefile free of charge from Statistics Austria upon request [32]; see the following website for details (only available in German language): https://www.sozialministerium.at/Themen/Gesundheit/Gesundheitssystem/Gesundheitsberichte/ (from here click on “Österreichische Gesundheitsbefragung 2014”).

## Abbreviations

DIF: Differential item functioning
CFA: Confirmatory factor analysis
CFI: Comparative fit index
df: Degrees of freedom
EQ-5D: European quality of life index version 5D
HRQOL: Health-related quality of life
ISCED: International Standard Classification of Education
MIMIC: Multiple indicators multiple causes
MLR: Robust maximum likelihood
RMSEA: Root Mean Square Error of Approximation
TLI: Tucker-Lewis-Index
SF-36: Short Form (36) Health Survey
SRMR: Standardized root mean square residual
WHOQOL: World Health Organization Quality of Life questionnaire
WHOQOL-BREF: World Health Organization Quality of Life questionnaire (short version)
