# Supplementary material for: Assessment of quality of life in individuals with chronic headache. Psychometric properties of the WHOQOL-BREF
Source: BMC Neurol. 2020 Jul 3;20:267. doi: 10.1186/s12883-020-01845-7 (PMC7333387; doi:10.1186/s12883-020-01845-7)
Supplement: Supplementary file 1 — Additional file 1 Correlations between the 24 WHOQOL-BREF items (individuals with self-reported chronic headache residing in Austria, Austrian Health Interview Survey, 2013–2015, n = 963) [file 12883_2020_1845_MOESM1_ESM.doc]

Appendix 1: Correlations between the 24 WHOQOL-BREF items (individuals with self-reported chronic headache residing in Austria, Austrian Health Interview Survey, 2013-2015, n=963)

|  | Item 3 | Item 4 | Item 5 | Item 6 | Item 7 | Item 8 | Item 9 | Item 10 | Item 11 | Item 12 | Item 13 | Item 14 | Item 15 | Item 16 | Item 17 | Item 18 | Item 19 | Item 20 | Item 21 | Item 22 | Item 23 | Item 24 | Item 25 | Item 26 |
| --- | --- | --- | --- | --- | --- | --- | --- | --- | --- | --- | --- | --- | --- | --- | --- | --- | --- | --- | --- | --- | --- | --- | --- | --- |
| Item 3 | 1 |  |  |  |  |  |  |  |  |  |  |  |  |  |  |  |  |  |  |  |  |  |  |  |
| Item 4 | 0.64 | 1 |  |  |  |  |  |  |  |  |  |  |  |  |  |  |  |  |  |  |  |  |  |  |
| Item 5 | -0.41 | -0.41 | 1 |  |  |  |  |  |  |  |  |  |  |  |  |  |  |  |  |  |  |  |  |  |
| Item 6 | -0.31 | -0.32 | 0.61 | 1 |  |  |  |  |  |  |  |  |  |  |  |  |  |  |  |  |  |  |  |  |
| Item 7 | -0.23 | -0.26 | 0.47 | 0.51 | 1 |  |  |  |  |  |  |  |  |  |  |  |  |  |  |  |  |  |  |  |
| Item 8 | -0.29 | -0.33 | 0.55 | 0.55 | 0.55 | 1 |  |  |  |  |  |  |  |  |  |  |  |  |  |  |  |  |  |  |
| Item 9 | -0.18 | -0.22 | 0.35 | 0.31 | 0.32 | 0.41 | 1 |  |  |  |  |  |  |  |  |  |  |  |  |  |  |  |  |  |
| Item 10 | -0.40 | -0.41 | 0.57 | 0.50 | 0.48 | 0.53 | 0.26 | 1 |  |  |  |  |  |  |  |  |  |  |  |  |  |  |  |  |
| Item 11 | -0.17 | -0.19 | 0.37 | 0.47 | 0.30 | 0.35 | 0.19 | 0.41 | 1 |  |  |  |  |  |  |  |  |  |  |  |  |  |  |  |
| Item 12 | -0.27 | -0.30 | 0.42 | 0.38 | 0.33 | 0.35 | 0.28 | 0.36 | 0.32 | 1 |  |  |  |  |  |  |  |  |  |  |  |  |  |  |
| Item 13 | -0.28 | -0.29 | 0.38 | 0.37 | 0.31 | 0.34 | 0.31 | 0.32 | 0.27 | 0.48 | 1 |  |  |  |  |  |  |  |  |  |  |  |  |  |
| Item 14 | -0.23 | -0.21 | 0.44 | 0.41 | 0.25 | 0.36 | 0.28 | 0.42 | 0.33 | 0.42 | 0.43 | 1 |  |  |  |  |  |  |  |  |  |  |  |  |
| Item 15 | -0.50 | -0.50 | 0.56 | 0.45 | 0.38 | 0.47 | 0.28 | 0.49 | 0.31 | 0.40 | 0.43 | 0.39 | 1 |  |  |  |  |  |  |  |  |  |  |  |
| Item 16 | -0.33 | -0.35 | 0.43 | 0.38 | 0.33 | 0.38 | 0.21 | 0.46 | 0.29 | 0.30 | 0.27 | 0.31 | 0.40 | 1 |  |  |  |  |  |  |  |  |  |  |
| Item 17 | -0.43 | -0.46 | 0.55 | 0.47 | 0.43 | 0.50 | 0.26 | 0.59 | 0.32 | 0.36 | 0.32 | 0.38 | 0.59 | 0.54 | 1 |  |  |  |  |  |  |  |  |  |
| Item 18 | -0.48 | -0.51 | 0.56 | 0.47 | 0.47 | 0.48 | 0.28 | 0.55 | 0.28 | 0.40 | 0.32 | 0.35 | 0.61 | 0.51 | 0.74 | 1 |  |  |  |  |  |  |  |  |
| Item 19 | -0.22 | -0.27 | 0.49 | 0.57 | 0.43 | 0.45 | 0.25 | 0.48 | 0.53 | 0.33 | 0.25 | 0.38 | 0.36 | 0.44 | 0.51 | 0.50 | 1 |  |  |  |  |  |  |  |
| Item 20 | -0.21 | -0.20 | 0.41 | 0.46 | 0.34 | 0.35 | 0.23 | 0.35 | 0.37 | 0.32 | 0.28 | 0.30 | 0.32 | 0.37 | 0.43 | 0.38 | 0.57 | 1 |  |  |  |  |  |  |
| Item 21 | -0.22 | -0.21 | 0.36 | 0.37 | 0.27 | 0.32 | 0.20 | 0.30 | 0.28 | 0.28 | 0.15 | 0.23 | 0.26 | 0.31 | 0.35 | 0.32 | 0.34 | 0.42 | 1 |  |  |  |  |  |
| Item 22 | -0.21 | -0.18 | 0.34 | 0.35 | 0.26 | 0.32 | 0.27 | 0.29 | 0.25 | 0.26 | 0.25 | 0.29 | 0.32 | 0.26 | 0.34 | 0.32 | 0.33 | 0.40 | 0.40 | 1 |  |  |  |  |
| Item 23 | -0.16 | -0.15 | 0.31 | 0.30 | 0.21 | 0.29 | 0.34 | 0.29 | 0.24 | 0.39 | 0.27 | 0.29 | 0.25 | 0.20 | 0.31 | 0.30 | 0.33 | 0.35 | 0.30 | 0.39 | 1 |  |  |  |
| Item 24 | -0.18 | -0.16 | 0.32 | 0.29 | 0.26 | 0.27 | 0.22 | 0.32 | 0.20 | 0.34 | 0.30 | 0.33 | 0.28 | 0.26 | 0.33 | 0.30 | 0.27 | 0.30 | 0.29 | 0.32 | 0.41 | 1 |  |  |
| Item 25 | -0.13 | -0.10 | 0.23 | 0.25 | 0.18 | 0.19 | 0.17 | 0.22 | 0.17 | 0.33 | 0.26 | 0.27 | 0.19 | 0.18 | 0.28 | 0.24 | 0.22 | 0.25 | 0.23 | 0.29 | 0.32 | 0.45 | 1 |  |
| Item 26 | 0.28 | 0.35 | -0.44 | -0.49 | -0.43 | -0.46 | -0.22 | -0.48 | -0.41 | -0.36 | -0.27 | -0.32 | -0.34 | -0.40 | -0.44 | -0.43 | -0.51 | -0.38 | -0.28 | -0.32 | -0.29 | -0.27 | -0.17 | 1 |
